# Laser activatable perfluorocarbon bubbles for imaging and therapy through enhanced absorption from coupled silica coated gold nanoparticles†

**DOI:** 10.1039/d0ra08009h

**Published:** 2021-01-29

**Authors:** Donald A. Fernandes, Sila Appak-Baskoy, Elizabeth Berndl, Michael C. Kolios

**Affiliations:** Department of Chemistry & Biology, Ryerson University Toronto ON M5B 2K3 Canada donald.fernandes@ryerson.ca; Institute for Biomedical Engineering, Science and Technology (iBEST), A Partnership Between Ryerson University and St. Michael's Hospital Toronto ON M5B 1T8 Canada; Keenan Research Centre for Biomedical Science of St. Michael's Hospital Toronto ON M5B 1T8 Canada; Department of Physics, Ryerson University Toronto ON M5B 2K3 Canada mkolios@ryerson.ca

## Abstract

Nanoparticles have extensively been used for cancer therapy and imaging (*i.e.*, theranostics) using various imaging modalities. Due to their physical and chemical properties (*e.g.*, absorption, fluorescence, and magnetic properties) they have been used for image guided therapy for cancer treatment monitoring. There are various limitations that make many theranostic agents unable to be used for the extended periods of time required for enhancing theranostic capabilities. Some of these are due to inherent characteristics (*e.g.*, change and/or breakdown of structure) present upon continuous irradiation and others are due to environmental (*i.e.*, physiological) conditions that can lead to physical instability (*i.e.*, in terms of size) affecting the amount of particles that can accumulate at the target site and the overall contrast that can be achieved. In this study, perfluorohexane (PFH) nanoemulsions (NEs) were synthesized with silica coated gold nanoparticles (PFH-NEs-scAuNPs) in order to give both stable and enhanced signals for cancer imaging by increasing vaporization of the emulsions into bubbles through the process of optical droplet vaporization (ODV). The resulting perfluorohexane bubbles could be imaged using nonlinear ultrasound (NL US) which significantly increases the signal to noise ratio due to the nonlinear scattering properties of oscillating bubbles. The NL US signals from PFH bubbles were found to be more stable compared to conventional bubbles used for contrast imaging. In addition, the vaporization of PFH NEs into bubbles was shown to cause significant cancer cell death reflecting the theranostic capabilities of the formed PFH bubbles. Since cell death is initiated with laser excitation of PFH-NEs-scAuNPs, these nanoparticles can specifically target cancer cells once they have accumulated at the tumor region. Due to the type of theranostic agent and imaging modality used, the PFH-NEs-scAuNPs can be used to provide higher specificity compared to other agents for locating the tumor region by minimizing tissue specific signals while at the same time being used to treat cancer.

## Abstract 2

PFH-NEs from PFH-NEs-scAuNPs can vaporize upon laser excitation leading to formation of PFH bubbles that can be used for contrast enhanced US imaging and therapy.

## Graphical abstract

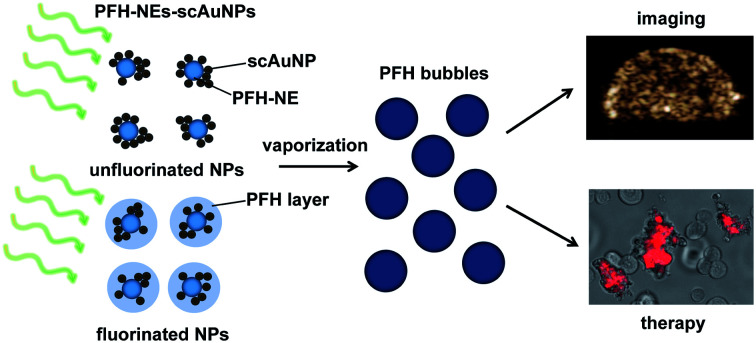

## 1. Introduction

Nanotechnology is a very fast growing and interdisciplinary field bringing together knowledge from chemistry, biology, physics and engineering. It has found many applications in industries such as agriculture^1^ and health care,^2^ the latter of which it is showing considerable potential. Research has expanded substantially in the development of various nanoparticles that can be used both for therapy and imaging of various diseases.^3–6^ An important class of nanoparticles are those made of inorganic material (*e.g.*, metal, metal oxides, semiconductors, silica) as their unique electric, magnetic and plasmonic properties enable them to destroy cancerous cells at the same time as being used to monitor tumor growth/regression through imaging.^7,8^ For example, iron based nanoparticles can induce hyperthermia in tissue through heat generation when under a magnetic field^9^ and/or be used as contrast agents for magnetic resonance imaging (MRI).^10^ Semiconductor nanoparticles such as quantum dots use their energy band gaps for fluorescence imaging with an alloy core encapsulated in an insulating inorganic shell to enhance their quantum yield.^11^ Due to their narrow fluorescence emission and broad absorption spectra, multiple quantum dots can be excited at a time for *in vivo* imaging of multiple components in biological systems.^12^ Such different types of nanoparticles show great potential for biomedical applications. Having said this, there continues to be a need for different methods/techniques and technologies for imaging and synthesizing nanoparticles that can greatly enhance specific target signals (*i.e.*, from tumor) while minimizing background signals (*i.e.*, from tissue). The NPs and imaging modality at the same time should allow for greater depths of imaging in tissue compared to conventional imaging.

One kind of inorganic nanoparticle able to provide deeper imaging *in vivo* are gold nanoparticles, able to release non-radiative heat from localized surface plasmon resonance, a phenomenon in which electrons oscillate coherently in resonance with the incident electromagnetic wave at a specific frequency. In most cases for deeper penetration and imaging *in vivo*, the frequency of oscillation is shifted from the visible to the near-infrared (NIR) region by increasing the aspect ratio of the nanoparticles^13^ and/or aggregation through the interparticle coupling effect.^14^ Both methods have been shown to enhance NIR absorption, with photothermal properties from absorption used for destroying cancer cells.^15–17^ A popular imaging modality used to detect signals from nanoparticles is photoacoustic (PA) imaging.^18,19^ Taking advantage of the absorption properties of nanoparticles and the resulting pressure generated upon optical excitation, PA imaging is able to distinguish the regions of nanoparticle accumulation for monitoring of cancer tumor therapy. Compared to other imaging modalities (*i.e.*, fluorescence and optical coherence tomography (OCT)), PA imaging can provide greater spatial resolution and deeper tissue penetration due to detection of ultrasonic signals, which attenuate less compared to visible electromagnetic waves.^20^ In our previous work we synthesized perfluorohexane nanoemulsions (PFH-NEs) that were able to give photoacoustic signals through the intrinsic near-infrared absorption properties of the fluorosurfactant shell of particles.^21^ The photoacoustic signals detected were from the vaporization of PFH-NEs into PFH bubbles due to conversion of the volatile PFH liquid into gas through the process of optical droplet vaporization (ODV).^22^ Coupling PFH-NEs with silica coated gold nanoparticles (scAuNPs) was shown to further increase PA signals in tissue-mimicking phantoms^23^ due to the enhanced absorption from scAuNPs surrounding the NEs. However even with the ability to provide strong acoustic signals, when using photoacoustic imaging *in vivo*, the background signal from blood and other tissue chromophores can reduce the contrast between tissues in which PFH-NEs-scAuNPs have accumulated and the surrounding tissues, even after spectral unmixing is applied. An approach with a greater target-to-background ratio is needed.

**Scheme 1:**
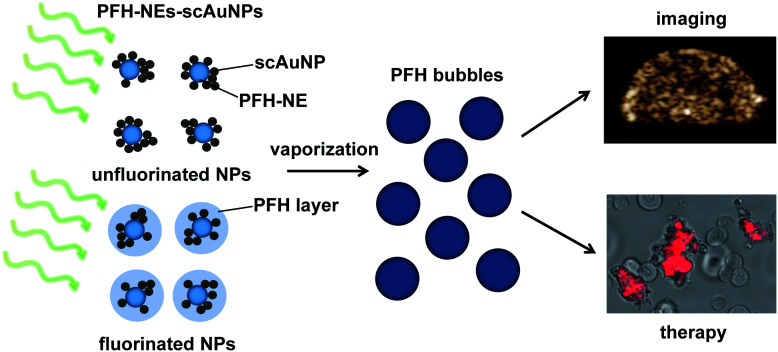
PFH-NEs-scAuNPs for theranostics. PFH-NEs from PFH-NEs-scAuNPs can vaporize upon laser excitation leading to formation of PFH bubbles that can be used for contrast enhanced US imaging and therapy (shown by propidium iodide labelling of nonviable cancer cells).

An imaging modality that provides high signal to noise for bio-imaging is contrast enhanced ultrasound (CEUS) imaging.^24^ CEUS imaging specifically detects signals from the contrast agent (*i.e.*, bubbles) targeting the region, suppressing unnecessary signals from background tissue. This is because signals from CEUS imaging are from nonlinear bubble subharmonic and superharmonic scattering,^25^ providing a highly specific signal confined to the location of the contrast agent (*i.e.*, bubbles). In this work we show that coupling scAuNPs to PFH-NEs can be used to give strong CEUS signals after laser induced vaporization of NEs into PFH bubbles through ODV (Scheme 1), with ODV and CEUS imaging demonstrated *in vivo*. The strong absorption properties of scAuNPs combined with its proximity with PFH-NEs can lead to efficient droplet vaporization into PFH bubbles with CEUS signals comparable and more stable than common commercial ultrasound contrast agents (*i.e.*, Definity microbubbles). The bimodal imaging capability of PFH-NEs-scAuNPs for CEUS and photoacoustic imaging^23^ can complement each other and increase treatment success by efficiently locating the tumor (using photoacoustics), and then imaging treated areas where signals are greatest (using CEUS). The imaging feedback would facilitate altering treatment conditions and dose, if required. The approach is similar to other important nanoparticle systems where CEUS is combined with other imaging modalities where the conversion of perfluorocarbon nanoparticles into bubbles upon laser excitation can be used to induce cancer cell death.^26,27^ The agents' ability to be used for deep tumor treatment and imaging by absorbing penetrating NIR light and the ability to provide ultrasound signals specifically from PFH bubbles make them highly advantageous over other theranostic agents. Moreover, compared to other nanoparticles, the PFH-NEs-scAuNPs developed have very good biocompatibility, enabling higher concentrations for improving theranostics. The nanoparticles can be easily coupled without the requirement of covalent linkage, minimizing the synthesizing steps and can be applied to a broad range of nanoparticles where theranostic capability can be provided through simple electrostatic interactions.

## 2. Materials and methods

### 2.1. Synthesis of PFH-NEs, scAuNPs and PFH-NEs-scAuNPs

To synthesize nanoemulsions, microemulsions were first made by mixing for 1 minute (2700 rpm using vortexer) a solution containing 600 μL perfluorohexane (PFH) (1100-2-07, Synquest Laboratories), 150 μL of Zonyl FSP fluorosurfactant (with anionic phosphate group) (09988, Sigma-Aldrich) and 4250 μL of Milli-Q water. A sonicator (Digital Model 250, Branson sonicator) was then used for 2 minutes using 10 seconds on/20 seconds off cycles at 4 °C in an ice water bath (20 kHz energy, 20% amplitude). To make gold nanoparticles, the sodium citrate reduction method^28^ was used by adding chloroauric acid (HAuCl_4_) while vigorously stirring sodium citrate in Milli-Q water at boiling. A coating of silica was added to gold nanoparticles by adding (3-aminopropyl)trimethoxysilane (APTMS) with a sodium silicate solution.^29^ The silica thickness of these nanoparticles were found to be around 10 nm with a 5 nm gold core sphere, to give a total size of 25 nm and absorption in the visible electromagnetic region^23^ (ESI Fig. S1a–c†). All concentrations for scAuNPs used for experiments were determined by weight of the gold nanoparticles only. Fluorinated scAuNPs (using 2 mL nanoparticle solution at 0.5 OD_max_ absorbance in visible electromagnetic region) in methanol (34860-1L-R, Sigma Aldrich) were made by mixing for 24 hours a solution of 1*H*,1*H*,2*H*,2*H*-perfluorodecyltriethoxysilane (PFDTES) (80 μL) (658758-25G, Sigma-Aldrich), ammonium hydroxide (70 μL) (320145-500ML, Sigma-Aldrich) and scAuNPs.^30,31^ To remove excess silica and unreacted products after silica coating and fluorination, particles were repeatedly washed using centrifugation. To make PFH-NEs with scAuNPs, particles were either mixed together after first emulsification of PFH-NEs (referred to as unfluorinated nanoparticles or unfluor. NPs) or first fluorinating scAuNPs and then suspending in PFH (600 μL) and adding Zonyl FSP (150 μL) and MilliQ water (4250 μL) prior to vortexing and sonication (using above settings) (referred to as fluorinated nanoparticles or fluor. NPs). To achieve the required concentrations for nanoparticles, samples were diluted or concentrated (using centrifugal filters, UFC901024, Millipore Sigma) in terms of weight over volume of solution after determining the mass of solutions of PFH-NEs and scAuNPs.

### 2.2. Characterization of nanoparticles and bubbles

To determine the absorption spectra of unfluor. and fluor. NPs, a NanoDrop spectrophotometer (ThermoFisher Scientific) was used. The size of PFH-NEs with scAuNPs were characterized using an Archimedes Particle Metrology System (Malvern Panalytical), which can measure size of particles less than ∼1 μm. The size distributions of negatively buoyant unfluorinated and fluorinated nanoparticles were determined by assuming the particles had the density of perfluorohexane (1.68 g mL^−1^) given the much greater concentration of PFH-NEs used relative to scAuNPs. The size distributions represented for each type of nanoparticle were from more than 500 nanoparticles to obtain a representative size distribution, with size reported as mean ± standard deviation from each distribution. To characterize the PFH bubbles, unfluorinated or fluorinated samples (equivalent to 0.025 mg mL^−1^ solution of PFH-NEs) were placed in 60 mm × 15 mm polystyrene dishes (Sarstedt, 83.3901) before laser excitation using 680 nm wavelength using a preclinical Vevo LAZR commercial imaging system (FUJIFILM VisualSonics Inc.). Nanoemulsions from each sample were vaporized for 5 minute intervals at three different locations in the dish before being imaged using a ZOE Cell Imager (Bio-Rad). For all brightfield images a gain of 8, exposure time of 300 ms, and LED intensity of 40 was used. The average size (±standard deviation) for PFH bubbles from unfluorinated and fluorinated samples were determined from more than 100 bubbles (for each type of nanoparticle) and quantified using ImageJ. The morphology of scAuNPs and PFH-NEs-scAuNPs were determined using transmission electron microscopy (TEM, JEOL JEM-1200 electron microscope, beam energy 80 kV) immediately after placing a few drops of sample on a grid with the absorption spectra of scAuNPs determined using a Perkin Elmer Lambda 20 UV/vis spectrometer. To image nanoparticles in MCF-7 cancer cells, an Olympus CKX41 microscope was used with 10× phase contrast objective after 1.5 hours incubation with unfluorinated (0.6 mg mL^−1^ PFH-NEs with 0.09 μg mL^−1^ scAuNPs) and fluorinated nanoparticles (0.6 mg mL^−1^ PFH-NEs with 0.036 μg mL^−1^ fluorinated scAuNPs). Cells were first grown at a concentration of 125 000 cells per mL for 24 hours before incubation with particles. Images were created using the given red, green, blue intensities for representing signals, with brighter intensities representing nanoparticles from the lighter background.

### 2.3. *In vitro* nonlinear ultrasound (NL US) imaging

For contrast enhanced ultrasound (CEUS) imaging of perfluorohexane bubbles formed after laser excitation of unfluorinated and fluorinated samples (PFH-NEs plus scAuNPs), a Vevo LAZR imaging system was used (with LZ250 transducer using 18 MHz frequency). The nonlinear contrast mode of the Vevo LAZR uses amplitude modulation sequences to generate the images with significant background signal suppression. The nanoparticles were injected in a tissue mimicking phantom made using 10% w/v Type A gelatin (G2500, Sigma-Aldrich) and 2% v/v formaldehyde (252549, Sigma-Aldrich) by first inserting nanoparticles in ∼1 mm inclusions, followed by imaging PFH bubbles after 10 s vaporization at 680 nm (see ESI Fig. S2a† for the experimental setup). For the droplet to bubble conversion, a tunable (680–970 nm) Nd:YAG laser with laser fluence of 20 mJ cm^−2^, repetition rate of 20 Hz and pulse duration of 4–6 ns was used. For unfluorinated samples, concentrations of PFH-NEs used were 2.5, 5, 10 and 20 mg mL^−1^ with 0.375, 0.75, 1.5 and 3 μg mL^−1^ of silica coated gold nanoparticles, respectively. For fluorinated samples, concentrations of PFH-NEs used were 2.5, 5, 10 and 20 mg mL^−1^ with 0.15, 0.3, 0.6 and 1.2 μg mL^−1^ of fluorinated silica coated gold nanoparticles, respectively. For comparison, NL US signals from PFH bubbles were compared relative to signals without PFH bubbles (*i.e.*, Milli-Q water) to determine NL US signal enhancement. Furthermore, ultrasound signals were also analyzed after vaporization from PFH bubbles compared to before vaporization from PFH-NEs-scAuNPs using the Vevo LAZR (at 21 MHz central frequency using LZ250 transducer) with the time/spatial domain signals used to convert into amplitude spectra using an established method through fast Fourier transform of RF data.^32^ To determine the stability of PFH bubbles from particles, unfluorinated (5 mg mL^−1^ PFH-NEs with 0.75 μg mL^−1^ scAuNPs) and fluorinated nanoparticles (5 mg mL^−1^ PFH-NEs with 0.3 μg mL^−1^ fluorinated scAuNPs) were mixed with 10% Type A gelatin with 2% v/v formaldehyde before the NEs were vaporized directly after making inclusions at day 0, and after 24 (day 1) and 48 hours (day 2). Before acquiring the NL US signals from PFH bubbles, each inclusion with particles was excited for 10 seconds at 680 nm at each day using the Vevo LAZR commercial system. Between days inclusions were placed in a 37 °C water bath.

To analyze CEUS images from MCF-7 cancer cells that incorporated the nanoparticles, a 125 000 cells per mL solution was first incubated for 24 hours followed by incubation for 4, 24 and 48 hours with nanoparticles. For experiments in cells, a PFH-NEs concentration of 10 mg mL^−1^ and silica coated gold nanoparticle concentration of 1.5 μg mL^−1^ were used for unfluorinated samples and 0.6 μg mL^−1^ of fluorinated scAuNPs with perfluorohexane nanoemulsions (10 mg mL^−1^) for fluorinated samples. After incubation of the nanoparticles with cells at the above time points, cells were washed three times with PBS to remove any external particles prior to trypsinization using 0.05% trypsin–EDTA. To make cell inclusions, the cells were mixed with Type A gelatin to create inclusions on the surface with the same concentration of gelatin (10% w/v with 2% v/v formaldehyde) prior to measurements in a heated water bath at 37 °C (see ESI Fig. S2b† for the experimental setup). To create PFH bubbles for CEUS imaging, unfluorinated and fluorinated nanoparticles were excited for 10 seconds at 680 nm. For nonlinear ultrasound imaging, a gain of 35 dB was used, while a gain of 45 dB and 35 dB were used for linear B-mode ultrasound for NPs alone and in cells, respectively (35 dB dynamic range). As a control MCF-7 cells were incubated with scAuNPs only for 24 hours (1.5 μg mL^−1^) and imaged with the same experimental and NL imaging conditions previously mentioned for imaging in cell inclusions. To measure NL US signals from cell inclusions in tissue mimicking layers, hemoglobin (0.67 mg mL^−1^) (H7379, Sigma-Aldrich) and 20% intralipid (7 mg mL^−1^) (I141, Sigma-Aldrich) were used to mimic the optical and acoustic properties of breast tissue.^33–36^ The same experimental conditions were used as above for incubation of nanoparticles with cells after 24 hours. A PFH-NEs concentration of 125 mg mL^−1^ and silica coated gold nanoparticle concentration of 18.8 μg mL^−1^ were used for unfluorinated samples and 7.5 μg mL^−1^ of fluorinated scAuNPs with perfluorohexane nanoemulsions (125 mg mL^−1^) for fluorinated samples, for incubation with MCF-7 cells. All imaging of cells with nanoparticles were carried out at non-cytotoxic concentrations as measured by trypan blue viability test (viability above 90% for both types of nanoparticles).

To compare NL US signals from bubbles (formed from the nanoparticles by ODV) to those conventionally used for ultrasound imaging, Definity microbubbles (Lantheus Medical Imaging) at a concentration of 3.3% volume/volume were used and the same procedure followed for experiments and analysis. To activate Definity bubbles vials were shaken using the Vialmix activation system from the company using their defined instructions for 45 seconds. For all experiments involving Definity bubbles, only NL US imaging was applied with no prior laser exposure. The averages reported for nonlinear ultrasound signals are the mean ± standard deviation from three replicates from gray scale values from a 3 mm × 1 mm region within the inclusion (for nanoparticles alone and with cells). NL US signals from *in vitro* and *in vivo* experiments in channels and with cells were determined to be statistically significant compared to controls (*i.e.*, with no contrast agents or before vaporization of NEs) using two sample *t*-test (*p*-value < 0.05).

### 2.4. Cell viability studies from droplet vaporization

To determine the potential of nanoparticles to cause cancer cell death through stresses caused by vaporization, nanoparticles were mixed with 926 000 MCF-7 cells in suspension (in DMEM media with 10% FBS and 0.01 mg mL^−1^ insulin) in 35 mm polystyrene dishes (Thermo Scientific, 130184). Samples were irradiated using 680 nm wavelength for two intervals of 10 minutes each using the Vevo LAZR at a focus of 11 mm. The Vevo LAZR sends 5 ns pulses of light (20 mJ cm^−2^) at a repetition frequency of 20 Hz. Samples (cells plus nanoparticles) were mixed between the two intervals of excitation to ensure previously untreated cells could be treated. A final concentration of 10 mg mL^−1^ of PFH-NEs were used for both unfluorinated (with 1.5 μg mL^−1^ of scAuNPs) and fluorinated samples (with 0.6 μg mL^−1^ of fluorinated scAuNPs). As a control MCF-7 cells were treated with scAuNPs only (1.5 μg mL^−1^) with same laser excitation conditions as above. Propidium iodide (P3566, Thermo Fisher Scientific) was used to determine nonviable cells after treatment by incubating 10 μL of a solution of 1 mg mL^−1^ of propidium iodide with cells for 5 minutes prior to washing cells with media. To image cells, a ZOE fluorescent imager was used with excitation of 556/20 nm and emission filter of 615/61 nm with viability analyzed using a Vi-Cell XR Cell Viability Analyzer (Beckman Coulter). To measure viability, 600 μL samples containing treated cells were placed in sample cups before quantifying nonviable cells based on the brightness from trypan blue labelled cells (after removing any NPs and bubbles using repeated centrifugation at 100 × *g* for 5 minutes each multiple times). The percent viability is reported as mean ± standard deviation from three replicates.

To determine the ability of PFH-NEs-scAuNPs to cause cancer cell death upon laser excitation and their effectiveness in treating other cell types, NPs were directly mixed with PC-3 prostate cancer cells (grown using RPMI-1640 media with 10% FBS). Using 125 000 cells either a NEs concentration of 20 mg mL^−1^ with 3 μg mL^−1^ of silica coated gold nanoparticles (ScAuNPs) for unfluorinated samples or NEs concentration of 20 mg mL^−1^ with 1.2 μg mL^−1^ of scAuNPs for fluorinated samples were used. For each replicate, 50 μL of cell solution with NPs were placed on 35 mm × 10 mm polystyrene dishes (Falcon, Corning 353001) with NPs vaporized at 680 nm using the Vevo LAZR at different treatment times (4, 8 and 12 minutes) for which cancer cells were continuously treated. The viability of cells was then quantified using Vi-Cell XR Cell Viability Analyzer (Beckman), using trypan blue as the dye for nonviable cells. Viability was quantified as percent of control PC-3 cells (without any NPs and treatment with laser), to determine the percent of viable cells after laser treatment with NPs (after removing any NPs and bubbles using centrifugation at 100 × *g* for 5 minutes each for three times). Viability of non-treated cells, with laser only and with NPs only (for both unfluor. and fluor. NPs) at the above treatment times were above 90%. The viability (% of control cells) is reported as mean ± standard deviation from three replicates. Additionally, the sizes of treated cells were recorded to determine the extent of cell damage using the Vi-Cell XR Cell Viability Analyzer.

### 2.5. Proof of principle *in vivo* studies

For proof of concept *in vivo* experiments for NL US imaging, BALB/c mice (6 weeks old) were purchased from Charles River Laboratories for determining the imaging capability of PFH bubbles. Tumors were grown using 150 000 4T1 breast cancer cells injected subcutaneously in the hindlegs of 6.5 weeks old mice. Tumors were then allowed to grow for 10 days before injecting nanoparticles intravenously through tail vein. For imaging each tumor, a dose of 4 mg of NEs per gram weight of mouse was used using a solution of 480 mg mL^−1^ of PFH-NEs with 0.08 mg mL^−1^ scAuNPs for unfluorinated nanoparticles, and 480 mg mL^−1^ of PFH-NEs with 0.03 mg mL^−1^ scAuNPs for fluorinated nanoparticles. Doses used for nanoemulsions and nanoparticles for *in vivo* experiments were below those used in other *in vivo* experiments for imaging and therapy.^37–40^ Furthermore, *in vitro* tests with cells at these doses showed high viability (>90% using trypan blue viability assay) in order to minimize cytotoxic effects. For therapy, tumors were irradiated (after 1.5 hours incubation of NPs) with 680 nm laser excitation (20 mJ cm^−2^ at 680 nm for 5 min for each treatment and time point for each day) using the Vevo LAZR system before collecting NL US images using the scanner. Multiple 2D CEUS images were collected from each tumor using the scanner from the Vevo LAZR imaging system. For CEUS and linear US imaging and acquiring signals, a CEUS gain of 35 dB and linear US gain of 50 dB were used. Signals are from PFH bubbles from maximum averaged gray scale signals from different slices within tumors compared to signals before injection of nanoparticles or before vaporization of nanoemulsions for each day. The Vevo LAB software from Vevo LAZR (FUJIFILM VisualSonics Inc.) was used for analysis of *in vivo* signals from both types of nanoparticles. For determining the therapeutic effect from laser excitation of PFH-NEs-scAuNPs and PFH bubbles, volume measurements of tumors (mm^3^) were determined using the 2D slices of images (collected using the Vevo LAZR for tumors) using the Vevo LAB software. All mice were handled according to the protocol (SMH protocol 870) approved by St. Michael's Hospital Animal Care Committee and by the Canadian Council on Animal Care.

## 3. Results and discussion

### 3.1. Size and charge of nanoparticles

**Fig. 1:**
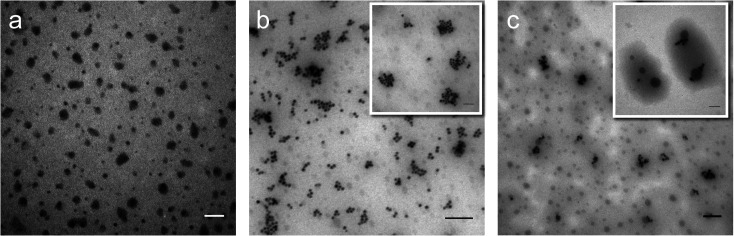
Morphology of PFH-NEs-scAuNPs. TEM images of PFH-NEs alone (with scale bar: 100 nm) (a) and with unfluorinated (b) and fluorinated scAuNPs (c) in Milli-Q water (scale bar: 200 nm; inset scale bar: 100 nm for both types of PFH-NEs-scAuNPs).

**Scheme 2:**
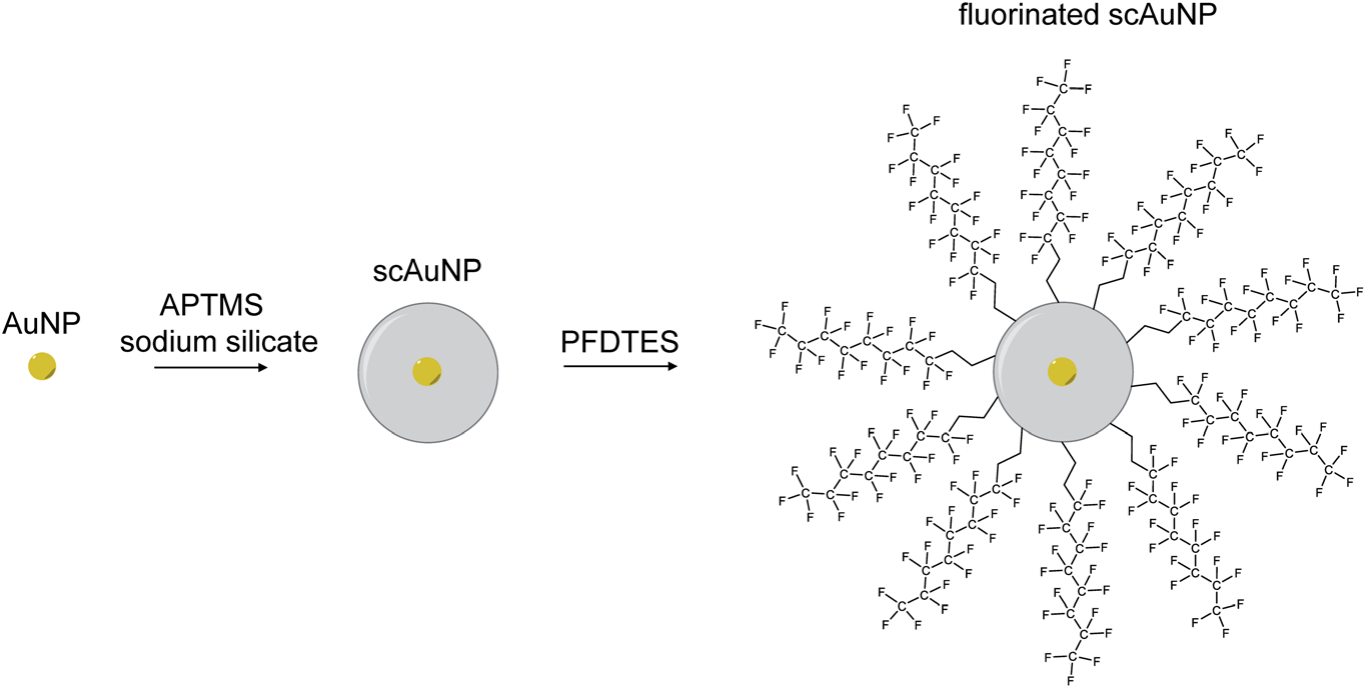
Silica coating and fluorination of scAuNPs. Schematic of process used to synthesize scAuNPs and fluorinate scAuNPs using (3-aminopropyl)trimethoxysilane (APTMS), sodium silicate and 1*H*,1*H*,2*H*,2*H*-perfluorodecyltriethoxysilane (PFDTES).

**Fig. 2:**
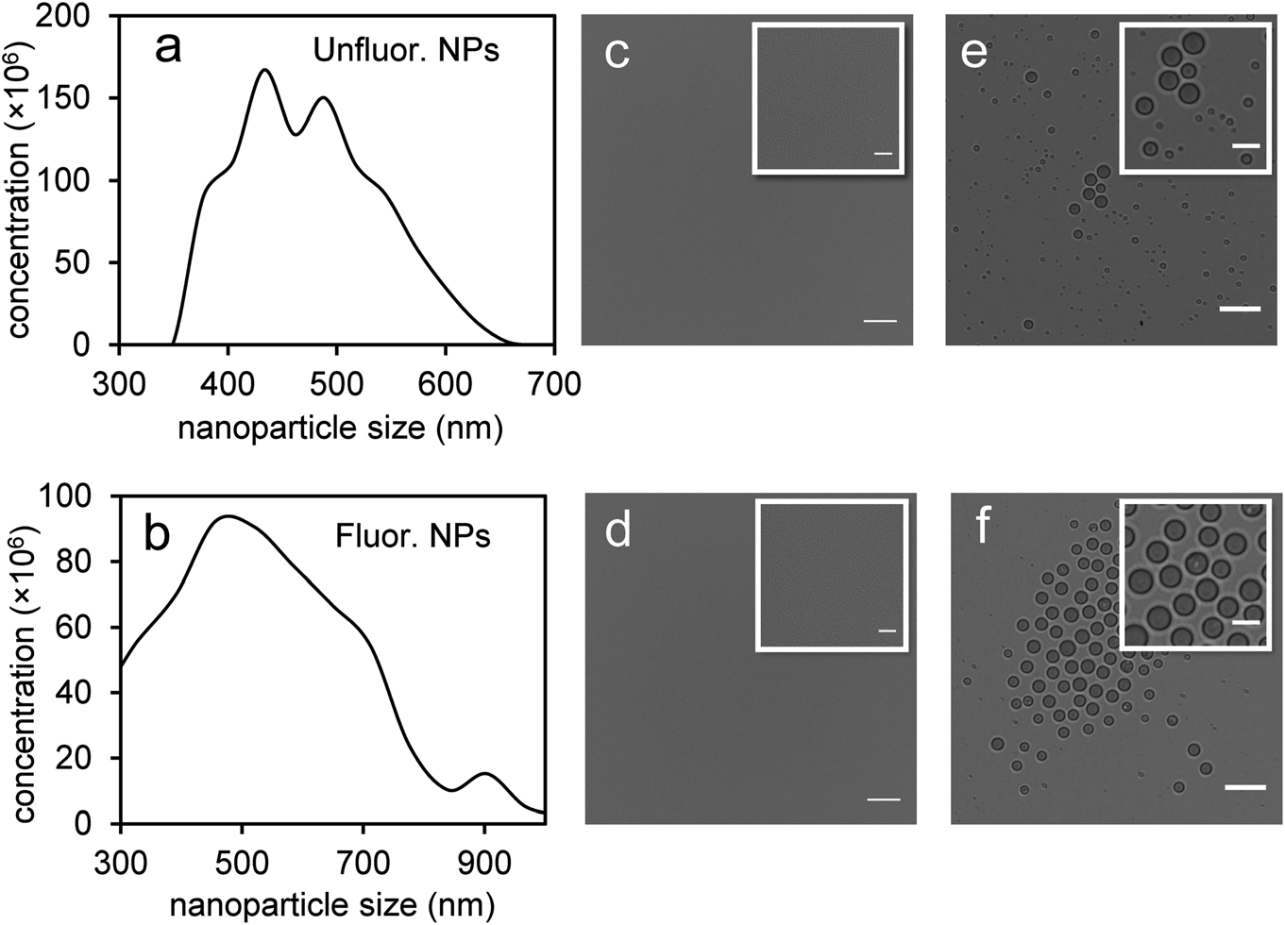
Characterization of size and morphology of PFH-NEs-scAuNPs and PFH bubbles. Size distributions (in terms of concentration in nanoparticles per mL) from Archimedes Particle Metrology System for unfluorinated (a) and fluorinated (b) samples (*x*-axis range slightly different due to differences in size of NPs). Images are from unfluorinated (c and e) and fluorinated (d and f) samples before (c and d) (scale bar: 50 μm, inset scale bar: 25 μm) and after (e and f) vaporization of NEs into bubbles (in Milli-Q water at 25 °C) (scale bar: 25 μm, inset scale bar: 10 μm).

Perfluorohexane nanoemulsions were synthesized previously using sonication and found to have a uniform size distribution around 50 nm (Fig. 1a)^21^ while scAuNPs were synthesized and fluorinated using APTMS, sodium silicate and PFDTES (see Materials and methods section 2.1, Scheme 2). Since the surface of PFH-NEs is made up of an anionic fluorosurfactant (Zonyl FSP), these NEs were found to be highly negatively charged (mean zeta potential of −72 ± 5 mV).^23^ Interaction with less negatively charged nanoparticles such as scAuNPs (mean zeta potential of −28 ± 3 mV),^23^ which absorb in the visible to near-infrared region (ESI Fig. S1b and c†) lead to the formation of clusters made up of both PFH-NEs and scAuNPs (PFH-NEs-scAuNPs) (Fig. 1b and c) most likely due to electrostatic attractions. The clustering of NPs leads to broadening and/or shift in the absorption spectra for unfluor. and fluor. NPs (ESI Fig. S3a and b†) towards the near-infrared region which is advantageous for exciting nanoparticles deeper in tissue for cancer imaging and therapy. This verifies that clusters made of PFH-NEs and scAuNPs are formed, with average cluster size for unfluorinated and fluorinated nanoparticles being 476 ± 63 nm and 510 ± 187 nm, respectively (Fig. 2a and b). The size of both unfluorinated and fluorinated nanoparticles were found to be similar to those reported previously,^23^ with size characterized using dynamic light scattering (DLS) and fluorescence cross-correlation spectroscopy (FCCS). By fluorescently labelling both the NEs and scAuNPs and using the latter technique (FCCS), it was determined that the PFH-NEs and scAuNPs are co-diffusing. The ability of these nanoparticles to form clusters is advantageous as the PFH-NEs can be vaporized into PFH bubbles after passing through larger sized endothelial gaps in the tumor vasculature,^41^ where they can be used for theranostics after accumulation at the tumor site.

**Fig. 3:**
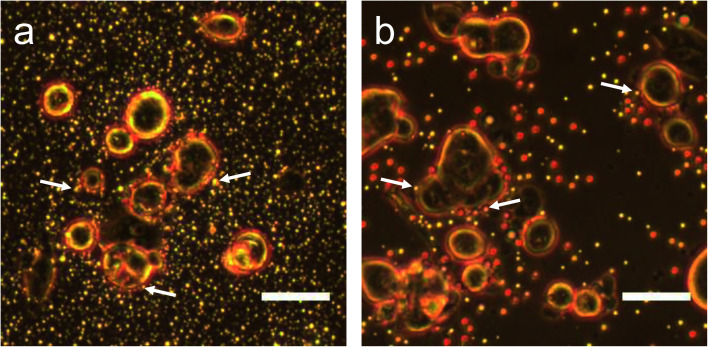
PFH-NEs-scAuNPs with MCF-7 cells. MCF-7 cells after 1.5 hours incubation (37 °C) with unfluorinated nanoparticles (a) and fluorinated (b) nanoparticles (scale bar: 50 μm) with white arrows pointing to nanoparticles/nanoparticle clusters located near the surface of cells. Images taken using phase contrast objective.

Since it was not possible to visualize unfluorinated and fluorinated nanoparticles due to the diffraction limit and low magnification of the light microscope (Fig. 2c and d), NEs were vaporized using laser pulses (680 nm wavelength) from the Vevo LAZR to detect the presence of larger microbubbles (Fig. 2e and f). Variations in the sizes of bubbles within samples for both unfluorinated and fluorinated samples (Fig. 2e and f) might be due to amount of fusion events between adjacent bubbles that occur during and/or directly after vaporization.^42,43^ The size of PFH bubbles formed from unfluorinated and fluorinated samples were 2.4 ± 0.5 μm and 7.3 ± 1.9 μm, respectively, determined from images captured 5 minutes after irradiation. The larger microbubbles formed are important for enhancing US scattering for locating tumor specific regions for imaging and therapy after nanoparticles have accumulated at the target site. To demonstrate that the NPs can efficiently target/internalize in cancer cells, NPs were imaged using phase contrast with MCF-7 cells. A significant number of highly scattering nanoparticles from both unfluorinated and fluorinated samples were localized at the membranes of cells (Fig. 3a and b), with the formation of significant amount of PFH bubbles also seen *in vitro* after brightfield illumination (due to localized heating from the continuous light) (ESI Fig. S4a and b†). These results suggest the potential of PFH-NEs-scAuNPs for CEUS imaging, by their ability to strongly attach to the membranes of cancer cells where the CEUS signals from bubbles can be used to specifically image tumors.

### 3.2. Nonlinear ultrasound (NL US) imaging of PFH bubbles

**Fig. 4:**
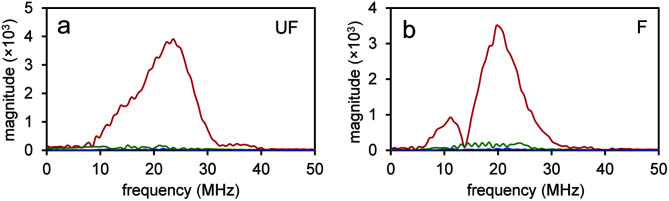
Amplitude spectra from nanoparticles and PFH bubbles. Frequency domain spectra from unfluor. (UF) (a) and fluor. (F) (b) samples from nanoparticles before vaporization (green) and after vaporization of PFH-NEs from PFH bubbles (red). For comparison signals from Milli-Q water only are shown (blue). Concentrations for unfluor. NPs was 2.5 mg mL^−1^ PFH-NEs with 0.37 μg mL^−1^ scAuNPs while for fluor. NPs it was 2.5 mg mL^−1^ PFH-NEs with 0.15 μg mL^−1^ scAuNPs.

Since the NEs from unfluorinated and fluorinated nanoparticles can be vaporized into PFH bubbles, the bubbles formed can be used for contrast enhanced ultrasound (CEUS) imaging. CEUS imaging (unlike ultrasound B-mode imaging) is a technique which is sensitive predominantly to the nonlinear scattering signals from bubbles (subharmonic and superharmonic generation from bubble scattering) and is able to provide high signal to noise compared to the linear scattering detected from the surrounding tissue. When ultrasound (US) contrast agents are used, CEUS is effective at locating the region the bubbles have accumulated, important in monitoring tumor size. In this application, the CEUS signals allow the spatial mapping of the locations where optical droplet vaporization (ODV) has occurred. To demonstrate this, the US signals were compared before (Fig. S5a and b†) and after vaporization of PFH-NEs (Fig. S5c and d†) showing signals from PFH bubbles being much greater due to the higher acoustic impedance mismatch from PFH gas (in bubbles). To further show that significantly higher acoustic signals can be achieved from PFH bubbles (compared to NPs) RF signals from the imaging system were analyzed. Higher acoustic magnitude values from PFH bubbles from both NPs were found in the 10–30 MHz region compared to NPs alone or Milli-Q water only due to the greater scattering from PFH bubbles required for signal enhancement in NL US imaging (Fig. 4a and b).

**Fig. 5:**
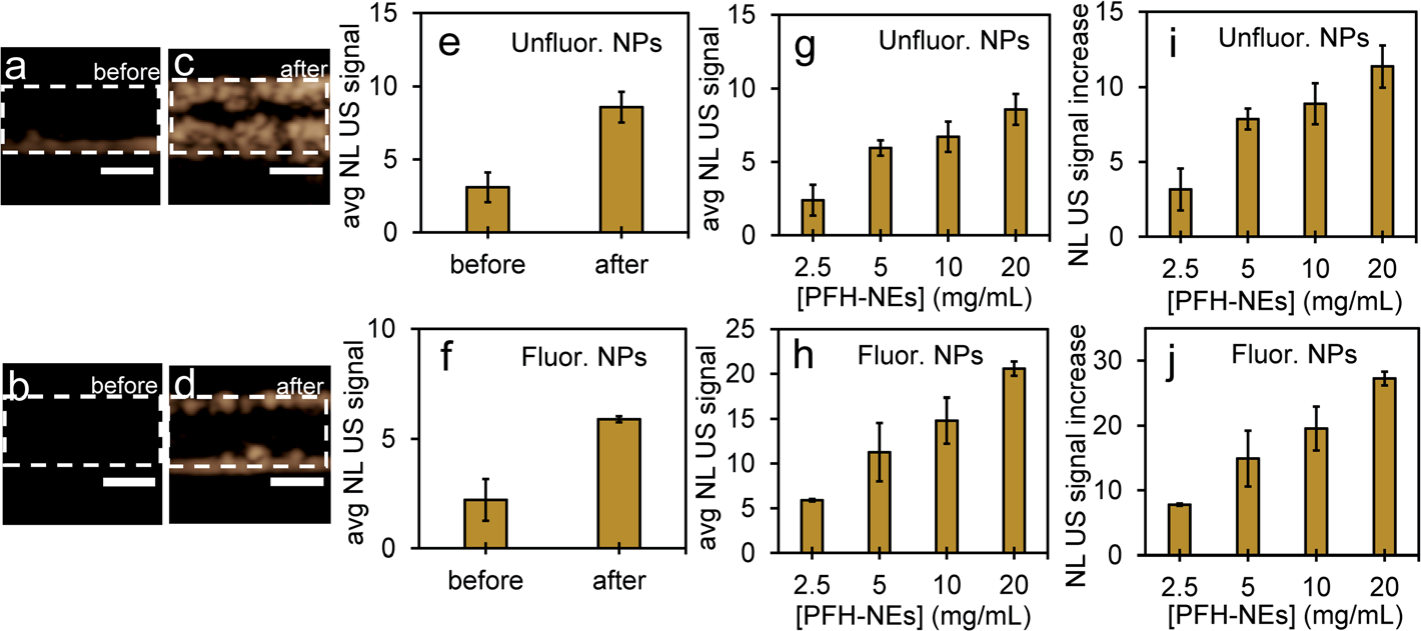
Nonlinear ultrasound imaging before and after vaporization from PFH-NEs-scAuNPs. Images before vaporization of NEs from unfluorinated and fluorinated nanoparticles (a and b) and after 10 s vaporization at 680 nm (c and d) (scale bar: 1 mm). After vaporization, the PFH bubbles formed had greater NL US signals (a.u.) from unfluor. (20 mg mL^−1^ NEs plus 3 μg mL^−1^ scAuNPs) (e) and fluor. samples (2.5 mg mL^−1^ NEs plus 0.15 μg mL^−1^ scAuNPs) (f) with corresponding NL US signals after vaporization into bubbles from different concentrations of unfluorinated (g) and fluorinated nanoparticles (h). All measurements were performed at 37 °C in ∼1 mm inclusions (outlined regions indicating where nanoparticles were placed before vaporization into PFH bubbles). Signals represent averaged gray scale values from three replicates measured from a rectangular region 3 mm × 1 mm in the phantom channel. For unfluorinated samples, concentrations of PFH-NEs used were 2.5, 5, 10 and 20 mg mL^−1^ with 0.375, 0.75, 1.5 and 3 μg mL^−1^ of silica coated gold nanoparticles, respectively. For fluorinated samples, concentrations of PFH-NEs used were 2.5, 5, 10 and 20 mg mL^−1^ with 0.15, 0.3, 0.6 and 1.2 μg mL^−1^ of fluorinated silica coated gold nanoparticles, respectively. For comparison NL US signals from NPs were compared to signals without PFH bubbles (*i.e.*, Milli-Q water in channel) by determining relative increases (i and j). Averaged NL US signal from Milli-Q water only was 0.76 ± 0.18. Each error bar represents standard deviation from three replicates.

Compared to before vaporization (Fig. 5a and b), CEUS signals from unfluorinated and fluorinated samples after laser excitation increased more than two times (Fig. 5c–f) due to strong nonlinear scattering from bubbles. Using both high and low concentrations of PFH-NEs and scAuNPs in both unfluorinated and fluorinated samples gave an increase in CEUS signals (Fig. 5a–d, g and h). There was also significant NL US signal enhancement (Fig. 5i and j) from PFH bubbles compared to signals without PFH bubbles (*i.e.*, Milli-Q water only, see ESI Fig. S6†) due to the greater amount of PFH bubbles formed with increasing concentration. These results show the ability of PFH bubbles to greatly enhance NL US signals specific to regions with PFH bubbles.

**Fig. 6:**
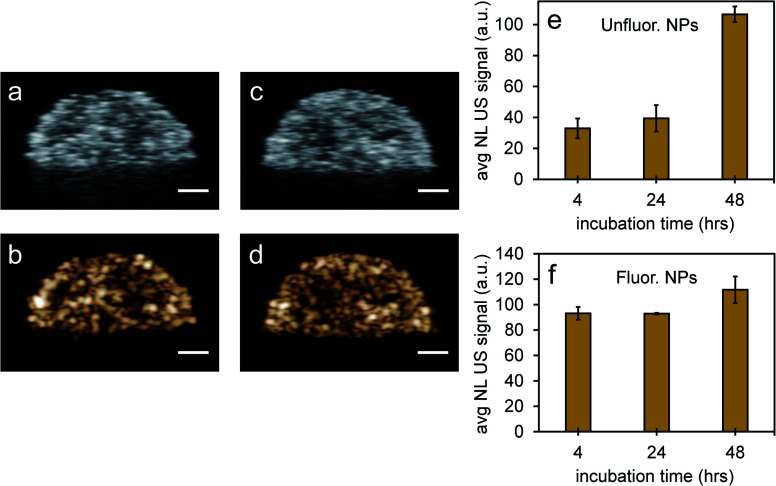
Nonlinear ultrasound imaging after vaporization of NEs from PFH-NEs-scAuNPs in MCF-7 cell inclusions. Simultaneous images of MCF-7 cells after vaporization of NEs from unfluor. (a and b) and fluor. NPs (c and d) from linear ultrasound (US) (a and c) and nonlinear ultrasound (NL US) (b and d) after 48 h incubation of NPs and 10 s vaporization at 680 nm (scale bar: 1 mm). The NL US signals for unfluor. (e) and fluor. NPs (f) from PFH bubbles were quantified after 10 s vaporization at 680 nm (at 37 °C) with signals representing averaged gray scale values from three replicates measured from a rectangular region 3 mm × 1 mm at the center of the inclusion. Each error bar represents standard deviation from three replicates.

Since it is important that nanoparticles are stable at physiological conditions, both types of nanoparticles were used to image PFH bubbles after vaporization in inclusions (Fig. 6a–d). Nanoparticles were incubated with MCF-7 breast cancer cells for 4, 24 and 48 hours before vaporization of NEs with 680 nm laser excitation. Unfluorinated samples showed a significant increase in CEUS signal after ODV and 48 hours incubation (Fig. 6e), while signals from fluorinated samples were stable with time (Fig. 6f). These signals were much greater compared to signals without NPs (ESI Fig. S7a–c†) and greatly enhanced due to vaporization of NEs as seen from experiments using contrast agents only (Fig. 5a–d). The differences in signals within the different incubation times and between samples might be due to differences in the internalization rates between the two types of particles. Since nanoparticles form clusters, the charge and morphology of particles can vary therefore affecting how fast cells can internalize these particles.^44–46^ Due to the different rates of cellular internalization, the appropriate NPs could be used for enhancing NL US imaging from PFH bubbles whether it be for short or long term imaging. Compared to our previous work with PFH-NEs only,^47^ the NL US signals from PFH-NEs-scAuNPs are greater after 48 hours incubation of nanoparticles possibly due to larger amount of bubbles formed after laser activation and more than five times greater compared to cells only (ESI Fig. S7b and c†).

**Fig. 7:**
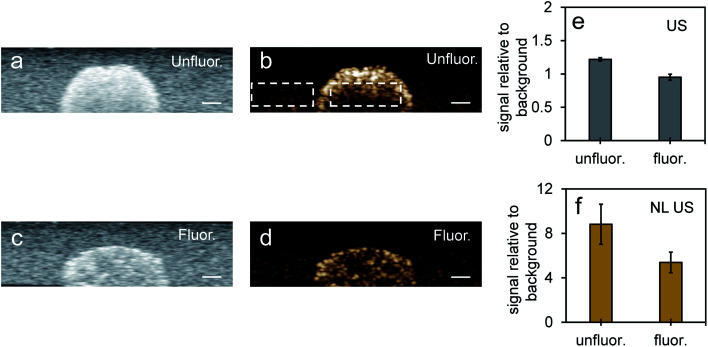
Imaging after vaporization of NEs from unfluor. and fluor. nanoparticles in MCF-7 cell inclusions in tissue mimicking phantoms. Simultaneous B-mode ultrasound (US) (a and c) and nonlinear ultrasound (NL US) (b and d) (scale bar: 1 mm) images of MCF-7 cells with PFH bubbles after 24 hours incubation and vaporization (10 s, 680 nm) from unfluorinated (a and b) and fluorinated nanoparticles (c and d). Corresponding signal (from inclusion) relative to background signal from tissue mimicking phantom for ultrasound (US) and nonlinear ultrasound (NL US) are shown in (e) and (f), respectively. All signals representing averaged gray scale values from three replicates measured from a rectangular region 3 mm × 1 mm at the center of the inclusion (relative to signal from a rectangular region 3 mm × 1 mm outside inclusion in the tissue mimicking layer, see Fig. 7b with rectangular regions for example). Each error bar represents standard deviation from three replicates.

Nonlinear signals from PFH bubbles from unfluor. samples were more than two times greater compared to scAuNPs only (at the same concentration of scAuNPs used for incubating with the unfluorinated sample) (ESI Fig. S8b and c†) and even more than five times greater comparing fluorinated NPs with scAuNPs only in cells (comparing Fig. 6f and S8c in ESI†) after 24 hours incubation. This indicates that the significant contributor in providing the NL US signals are the PFH bubbles which scatter US waves. When NL US signals were measured from laser activated bubbles in cancer cells within a tissue mimicking layer (with optical and acoustic properties of tissue)^33–36^ (Fig. 7a–d) NL US signals were more than five times greater compared to the background tissue mimicking layer (Fig. 7b, d and f). NL US signals compared to background tissue mimicking signals were much greater than conventional B-mode US (Fig. 7e and f) suggesting the ability of PFH bubbles to give better contrast for NL US imaging after vaporization of the NEs. These results show the ability of NL US imaging to provide higher contrast than B-mode ultrasound imaging by inhibiting strong tissue backscattering and thus enhancing the contrast-to-tissue ratio (CTR) due to the strong nonlinear responses of bubbles formed.

**Fig. 8:**
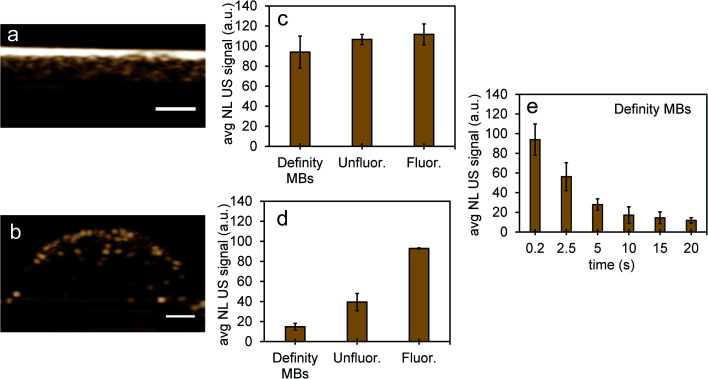
NL US signals from unfluor. and fluor. NPs after vaporization of NEs compared to Definity microbubbles. Nonlinear US images (a and b) and signals (c and d) from PFH bubbles from unfluor. and fluor. samples from MCF-7 cells after 48 h (c) and 24 h (d) incubation of NPs compared to signals from Definity microbubbles alone (c) and with MCF-7 cells after 24 h incubation (d) (using 3.3% v/v concentration) (all measurements at 37 °C) (scale bar: 1 mm). The stability of Definity microbubbles is shown in (e) using setup Fig. S2a in ESI.† Results shown in (c) compare signals from Definity bubbles (using setup Fig. S2a†) directly after activation of bubbles while results in (d) compare signals using setup Fig. S2b in ESI† with no laser exposure. All signals representing averaged gray scale values from three replicates measured from a rectangular region 3 mm × 1 mm at the center of the inclusion. Each error bar represents standard deviation from three replicates.

To compare NL US signals from bubbles created from nanoparticles by ODV to that of conventional microbubbles, the NL signals from Definity microbubbles were measured at a concentration (3.3% volume/volume) commonly used in previous cell studies, found to be both optimal and non-cytotoxic for cell experiments.^48–50^ When comparing signals in inclusions from Definity bubbles (Fig. 8a and b), NL US signals from bubbles from unfluor. and fluor. nanoparticles after 48 hours incubation with MCF-7 cells had very similar signal strengths (Fig. 8c) and had more than two times greater signals than from Definity bubbles with cells after 24 hours incubation and laser excitation (Fig. 8d). This suggests that PFH-NEs-scAuNPs can be used to significantly enhance the nonlinear ultrasound contrast at non-cytotoxic concentrations (ESI Fig. S9†) in cancer cells and that the addition of optical absorbers (*i.e.*, scAuNPs) leads to a decrease in the vaporization threshold of nanoparticles^51^ resulting in a greater amount of bubbles formed. Since most conventional bubble contrast agents are unstable, with dissolution (disassembly) occurring within few hours,^52^ unfluorinated and fluorinated nanoparticles can be used for long-term NL US imaging. Since nanoemulsions from these nanoparticles convert into bubbles only upon laser excitation they can have much longer physical stability compared to other contrast agents, and hence the bubbles formed from vaporization of NEs can be utilized for imaging for longer time periods. The longer stability of PFH bubbles after vaporization of NEs might be due to differences in composition for the shell (Zonyl FSP fluorosurfactant) and core (perfluorohexane), as the PFH bubbles may not be affected to the same extent by chemical and physical properties (such as surface tension, gas solubility and diffusivity)^52,53^ compared to other micro- and nanobubble contrast agents (as seen by stable or increasing signals in Fig. 6e and f compared to decreasing signals from bubbles in Fig. 8e).

**Fig. 9:**
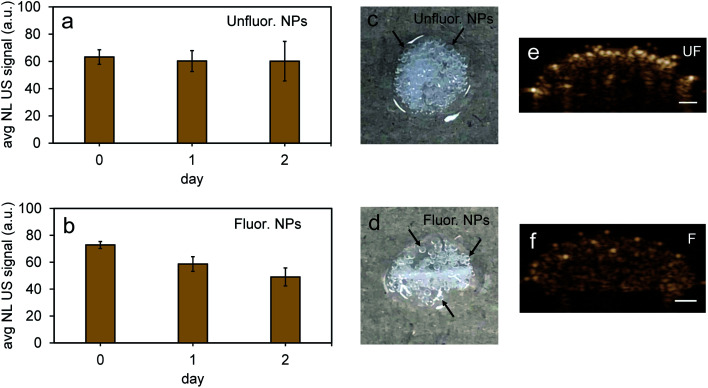
Stability of PFH bubbles from unfluorinated and fluorinated nanoparticles. Stability of nonlinear ultrasound (NL US) signals from PFH bubbles at day 0, 1 and 2 (at physiological temperature of 37 °C) for unfluorinated (UF) (a) and fluorinated (F) (b) PFH-NEs-scAuNPs in inclusions. Representative images from unfluorinated (c) and fluorinated nanoparticle inclusions (d) (∼4–5 mm) show the formation of visible PFH bubbles after 48 hours and 680 nm vaporization using the Vevo LAZR. Inclusions were irradiated for 10 seconds at 680 nm before measuring NL US signals at day 0, 1 and 2. NL US images from bubbles after vaporization of NEs from unfluorinated (UF) and fluorinated (F) nanoparticles are shown in (e) and (f), respectively (scale bar: 1 mm). All signals represent averaged gray scale values from three replicates measured from a rectangular region 3 mm × 1 mm at the center of the inclusion. Each error bar represents standard deviation from three replicates.

To further test the stability of PFH bubbles from unfluorinated and fluorinated nanoparticles, particles and bubbles were exposed to multiple laser pulses over a period of two days and the NL US signals measured after day 0, 1 and 2. PFH bubbles from both unfluorinated and fluorinated samples were very stable after 48 hours at physiological temperature and laser excitation (Fig. 9a–f). There were no significant changes in NL US signals from unfluorinated samples (Fig. 9a), with NL US signals decreasing 29% (compared to day 0) at day 2 from fluorinated samples (Fig. 9b). Comparing NL US signals, PFH bubbles from PFH-NEs-scAuNPs (Fig. 9c–f) have the potential to provide greater NL US signals compared to conventional US contrast bubbles used for imaging (*i.e.*, Definity microbubbles). Compared to Definity bubbles which have previously been used for comparison with extravascular US contrast agents,^54^ the PFH bubbles from nanoparticles are much more stable in terms of rate of NL US signal decay (Table S1†). Some primary factors influencing the high stability of PFH bubbles is the type of stabilizer used (in this case Zonyl FSP fluorosurfactant) in lowering interfacial tension, the lower solubility and diffusivity of perfluorohexane (compared to other gases used to make bubbles)^55^ which increases the dissolution time before PFH bubbles undergo collapse. The interfacial tension can be reduced by increasing the chain length of the encapsulating shell of bubbles which can extend stability of bubbles.^56,57^ The results show the high stability and specificity of PFH bubbles from nanoparticles at physiological conditions and the great potential of these nanoparticles as *in vivo* contrast agents for tumor monitoring during therapy.

### 3.3. Vaporization induced cell death from PFH-NEs-scAuNPs

**Fig. 10:**
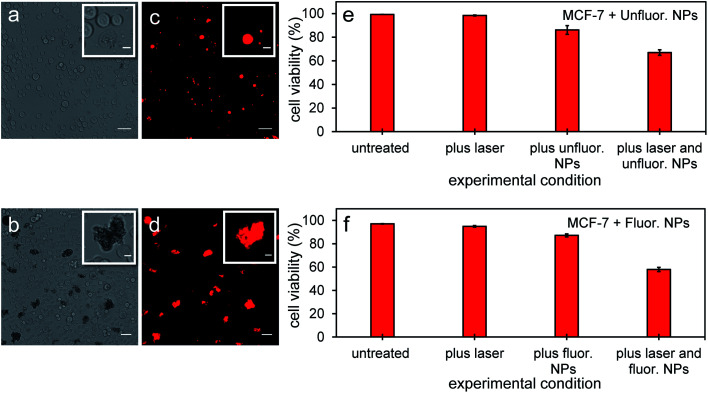
MCF-7 cells after vaporization of NEs from PFH-NEs-scAuNPs. Brightfield (a and b) and fluorescence (c and d) images from viable MCF-7 cells and those damaged by vaporized NEs from unfluor. (a and c) and fluor. nanoparticles (b and d) after 680 nm wavelength excitation. Damaged cells with compromised cell membranes are labelled with propidium iodide showing the location of nuclear content (DNA and RNA) in cells (c and d) with the corresponding cell viability (as percentage, %) from unfluor. (e) and fluor. sample (f) (scale bar: 50 μm). Insets in Fig. 10a–d show cells with membrane damage (labelled with cell permeable dye propidium iodide) from treatment with NPs with clearly distinguishable cell morphologies compared to untreated cells (scale bar: 10 μm). Smaller propidium iodide labelled nonviable cells could be seen under brightfield microscope but not clearly detected under fluorescence mode due to detection limit of the microscope. The cell viabilities reported represent averaged values with each error bar representing standard deviation from three replicates.

Since PFH NEs from unfluorinated and fluorinated samples can vaporize and have the potential to cause intracellular disruption due to the bubble expansion, the viability of MCF-7 cells was determined (directly after mixing cells with particles and treatment). Experiments from both types of particles showed a significant amount of cell death in breast cancer cells after exposing cells with nanoparticles and laser light. Significant changes in cell morphology and size were seen depending on the amount of NPs localized with cancer cells leading to different types of damage to their cell membranes compared to untreated cells (Fig. 10a and b). Membrane damage to cells, as assessed by the ability of propidium iodide to enter cells (Fig. 10c and d) is most likely from the cellular disruption during the conversion of the PFH-NEs into bubbles in the unfluorinated and fluorinated samples. To determine extent of cell death in the entire cell population, viability was quantified using an automated cell viability system (*i.e.*, Vi-Cell XR Cell Viability Analyzer) after laser treatment with NPs. Viability for unfluorinated and fluorinated samples were 67 ± 2% and 58 ± 2% (Fig. 10e and f) respectively, due to the greater amount of PFH bubbles formed from vaporization and was greater than when using NEs alone for laser treatment (viability 75 ± 7%).^21^ Since the viability of MCF-7 cells after laser exposure using the same concentration of scAuNPs (1.5 μg mL^−1^) as that from unfluorinated samples showed viability of 93 ± 1% (after laser excitation), this suggests that cell death is primarily from the formation of the PFH bubbles. Results show that the addition of scAuNPs with PFH-NEs, can not only be used to increase CEUS signals for imaging of tumors but also can lead to more cell death (10–20% decrease in viability) compared to when using PFH-NEs alone. Compared to other therapeutic nanoparticles, PFH-NEs-scAuNPs can provide immediate treatment without waiting for NPs to internalize in cancer cells. Their ability to provide significant stress induced damage only after accumulating at the tumor site (due to the enhanced permeability and retention of tumor vasculature)^41^ and after laser excitation within the irradiated region make these NPs advantageous, minimizing cellular damage in healthy tissue.

**Fig. 11:**
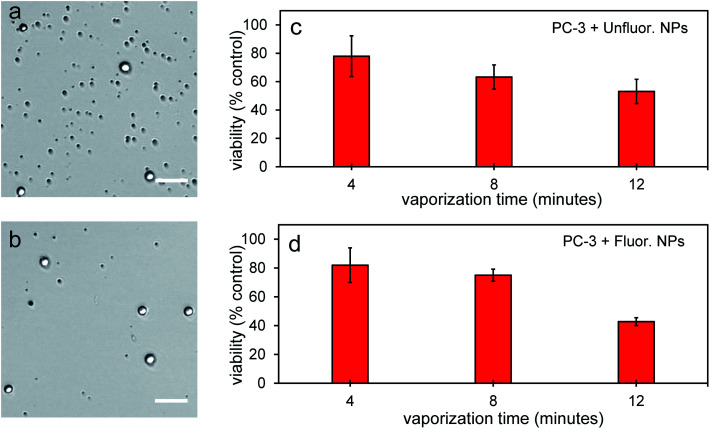
Cell viability from vaporization from PFH-NEs-scAuNPs in PC-3 cells. Brightfield images of cells from vaporization from unfluorinated (a) and fluorinated NPs (b) after 12 minutes laser excitation at 680 nm using Vevo LAZR system (scale bar: 50 μm). Significant cell death is seen and quantified after various times of laser excitation at 680 nm for both unfluor. (c) and fluor. NPs (d). In images viable cells are brighter than nonviable cells (darker) due to the uptake of the viability dye, trypan blue. Viability of non-treated cells, with laser only and with NPs only (for both unfluor. and fluor. NPs as well as scAuNPs alone) at the above treatment times were above 90%. The cell viabilities reported represent averaged values with each error bar representing standard deviation from three replicates.

To further determine the therapeutic capability of PFH-NEs-scAuNPs, cancer cells were treated with NPs with different laser treatment times. Viability was between 40–50% after 12 minutes laser treatment (Fig. 11a–d) with NPs showing the ability to cause significant cancer cell death for treatment of other cancer cell types (*i.e.*, prostate cancer cells). After treatment there was significant number of smaller nonviable cancer cells/cell fragments seen only after laser irradiation of NPs (Fig. 11a and b, S10a–d and S11a–f in ESI†) showing the ability of vaporization of NEs to cause significant cellular damage for therapy. The size of the smaller nonviable cells/cell fragments are approximately 4 μm (ESI Fig. S10b and d†) and very similar to the sizes seen when MCF-7 breast cancer cells were treated with nanoparticles and laser treatment (Fig. 10a–d). These results show the ability of vaporization of NEs to decrease tumor size by decreasing both cancer cell viability and size leading to overall lower number of viable cells for effective treatment outcomes. The cancer cell death efficiency is a result of both the physical stability of the nanoparticles and bubbles as well as the photo-stability of these contrast agents under continuous laser excitation. As significant cancer cell death can be achieved without drug loading, PFH-NEs-scAuNPs might be more advantageous for *in vivo* applications compared to other drug loaded theranostic agents which often have fast release rates of drugs during blood circulation which can lead to unintended consequences such as systemic toxicity from high drug accumulation in healthy tissues.^58^

### 3.4. *In vivo* stability of PFH-NEs-scAuNPs and PFH bubbles for theranostics

**Fig. 12:**
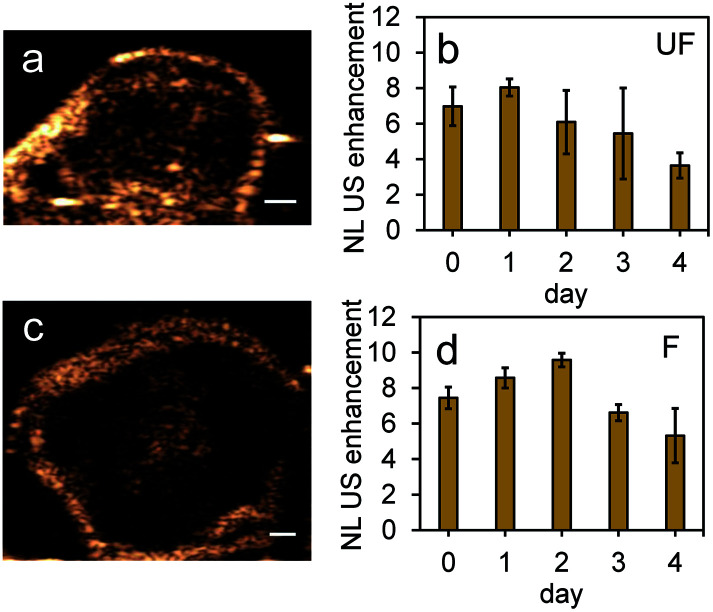
*In vivo* NL US images and signals from PFH-NEs-scAuNPs. Images and signals from unfluorinated (UF) (a and b) (day 4) and fluorinated (F) (c and d) nanoparticles (day 2). NL US images were captured after 5 minutes vaporization at 680 nm (a and c) from PFH bubbles using the Vevo LAZR (scale bar: 1 mm). NL US signal enhancement from bubbles from both unfluorinated (b) and fluorinated (d) nanoparticles are shown after 5 minutes vaporization for each day compared to signals before injection of the NPs. All signals (mean ± standard deviation) analyzed from *n* = 3 and *n* = 2 mice for experiments using unfluorinated and fluorinated nanoparticles, respectively. Signals at day 0 are from 1.5 hours after injection of NPs.

**Fig. 13:**
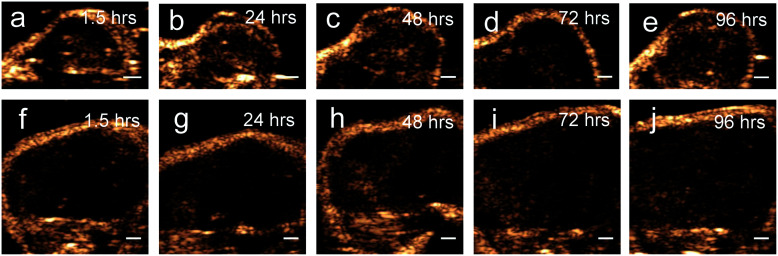
*In vivo* NL US images from PFH-NEs-scAuNPs. NL US images from PFH bubbles after 5 minutes vaporization (at 680 nm using Vevo LAZR) of PFH-NEs from unfluorinated (a–e) and fluorinated (f–j) nanoparticles after different time points after injection of NPs (scale bar: 1 mm).

**Fig. 14:**
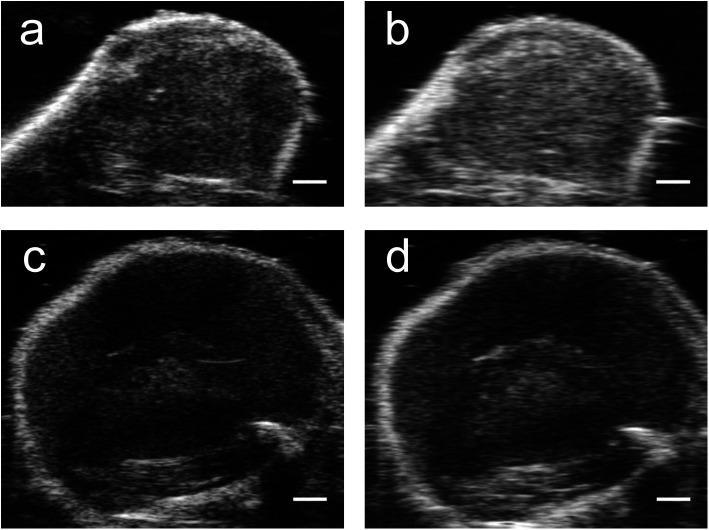
*In vivo* US images from PFH-NEs-scAuNPs. Images from unfluorinated (UF) (a and b) (day 4) and fluorinated (F) (c and d) nanoparticles (day 2). Linear US images were captured before (a and c) and after 5 minutes vaporization at 680 nm (b and d) from PFH bubbles using the Vevo LAZR (scale bar: 1 mm). Increase in linear B-mode US signals in images from bubbles from both unfluorinated and fluorinated nanoparticles are shown after 5 minutes vaporization compared to before 5 minutes vaporization for day 4 for unfluor. NPs and day 2 for fluor. NPs.

Results from the proof of principle *in vivo* experiments showed the presence of PFH bubbles (Fig. 12a and c) and that NL US signals from vaporization of NEs from PFH-NEs-scAuNPs were enhanced even after 4 days after injection of nanoparticles (Fig. 12b and d). Differences seen in NL US signals between days are likely due to different rates of accumulation of the two types of nanoparticles at the tumor region. NL US signal enhancement was highest at day 1 for unfluorinated NPs (Fig. 12b) and highest at day 2 for fluorinated NPs (Fig. 12d) when comparing signals to those from tumors with no nanoparticles (ESI Fig. S12b†). NL US signals were present *in vivo* due to both the stability at physiological conditions of NPs as well as the stability of PFH bubbles using both unfluorinated (Fig. 13a–e) and fluorinated nanoparticles (Fig. 13f–j). The long blood circulation time of nanoparticles might arise from the silica shell from scAuNPs near PFH-NEs as well as the morphology of PFH-NEs-scAuNPs, as in previous work the silica coating and shape in other NPs have been shown to be important for long term *in vivo* bioimaging and slow clearance and metabolism.^59,60^ The US results further show increases in linear (B-mode) ultrasound signals from the presence of NEs (Fig. 14a and c) that can vaporize upon laser irradiation into bubbles (Fig. 14b and d) with linear ultrasound enhancement seen till at least 4 days from injection of NPs. Results from *in vivo* experiments support those from *in vitro* experiments (Fig. 9a–f) showing the presence of PFH bubbles after laser excitation due to the stability of PFH-NEs-scAuNPs and PFH bubbles. Such high stability can enable long term imaging during cancer therapy. Furthermore laser excitation of NPs during the 4 day imaging period led to decrease in tumor volume at each day (compared to with laser exposure only) due to the ability of nanoparticles to cause cancer cell death through vaporization (ESI Fig. S13a–c†) showing the capability of using PFH-NEs-scAuNPs for enhanced theranostics and potential for clinical applications.

## 4. Conclusions

PFH-NEs-scAuNPs can be used to increase both nonlinear ultrasound signals and cell death due to the reduction of the vaporization threshold and energy required for converting PFH-NEs into bubbles (through optical droplet vaporization). This is accomplished by the addition of scAuNPs which can efficiently transfer energy to PFH-NEs to convert their liquid PFH core into the gas phase, creating greater amount of PFH bubbles. The NL US signals from PFH bubbles generated from vaporization of PFH-NEs are greater and more stable than common lipid-based bubbles used for medical imaging (*i.e.*, Definity). The signals from PFH bubbles were stable *in vivo* for at least four days, with future work involving experiments of biodistribution of nanoparticles for explaining the stability. The PFH-NEs-scAuNPs can simultaneously be used for imaging and therapy as theranostic agents as the PFH bubbles formed can create strong non-linear ultrasound signals and cause damage to cell membranes of cancer cells. The vaporization of NEs can be tuned (*i.e.*, by varying laser treatment time) to cause significant cancer cell death without the addition of any therapeutic agents. As the mechanism for cell death is through the stress induced by the vaporization of NEs, treatment using the NPs provides an effective alternative in inhibiting tumor growth, through permanent physical damage to cells. The ability to add optically absorbing agents (*i.e.*, scAuNPs) to nanoemulsions has the potential to be applied to other nanoparticle systems for enhancing theranostic outcomes and due to the characteristics of these theranostic agents can serve as a better non-invasive alternative compared to other nanoparticle-based techniques.

## Conflicts of interest

The authors declare they have no conflicts of interest.

## Supplementary Material

RA-011-D0RA08009H-s001
