# Supplementary material for: Laser activatable perfluorocarbon bubbles for imaging and therapy through enhanced absorption from coupled silica coated gold nanoparticles
Source: RSC Adv. 2021 Jan 29;11(9):4906–20. doi: 10.1039/d0ra08009h (PMC8694477; doi:10.1039/d0ra08009h)
Supplement: RA-011-D0RA08009H-s001 [file RA-011-D0RA08009H-s001.pdf]

Electronic Supplementary Material (ESI) for RSC Advances.
This journal is © The Royal Society of Chemistry 2021

Supplementary Information:

Laser activatable perfluorocarbon bubbles for imaging and therapy through enhanced absorption from coupled silica coated gold nanoparticles

Authors:

Donald A. Fernandes[a,b,c], Sila Appak-Baskoy[a,b,c], Elizabeth Berndl[b,c,d] and Michael C. Kolios[b,c,d]

Author Affiliations:

[a]Department of Chemistry & Biology, Ryerson University, Toronto, ON, M5B 2K3, Canada

[b]Institute for Biomedical Engineering, Science and Technology (iBEST), a partnership between Ryerson University and St. Michael's Hospital, Toronto, ON, M5B 1T8, Canada

[c]Keenan Research Centre for Biomedical Science of St. Michael's Hospital, Toronto, ON, M5B 1T8, Canada

[d]Department of Physics, Ryerson University, Toronto, ON, M5B 2K3, Canada

Supplementary Tables

Table S1. Rate of NL US signal decay as a measure of stability of PFH bubbles. As comparison rate of signal decay is provided for conventional bubbles used for US imaging *(i.e.*, Definity bubbles). Rates determined using linear regression and exponential fitting (using Figure 8 e and Figure 9 a and b). The coefficient of determination (R^2) for the fits were 0.94, 0.99, and 0.77 for Definity, fluor. and unfluor. NPs, respectively.

Sample	Rate of Signal Decay
Definity	−0.426/s
Unfluor. NPs	-17.63×10^{-6}/s
Fluor. NPs	-137.58×10^{-6}/s

Supplementary Figures

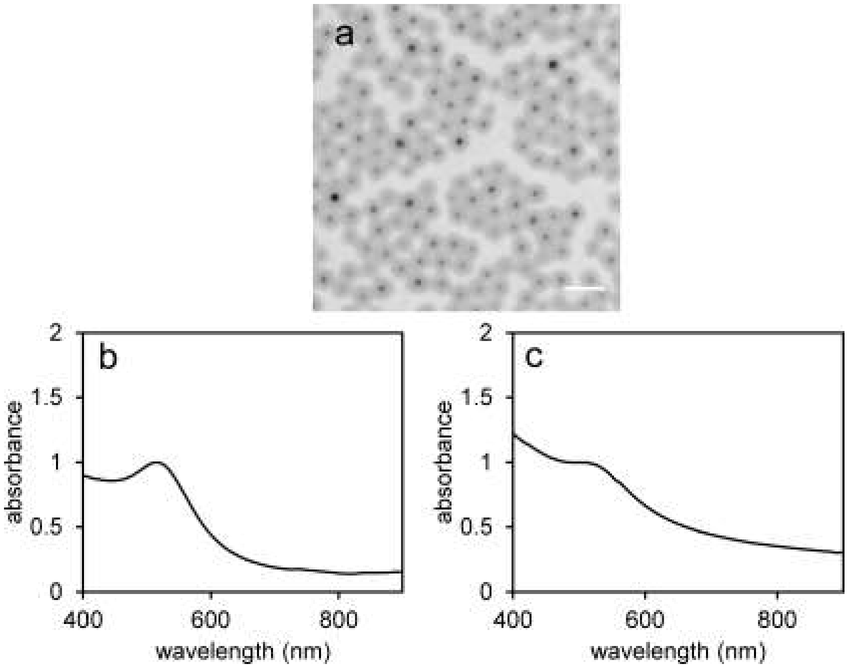

Figure S1. Characterization of scAuNPs. TEM image of scAuNPs showing 5 nm gold core with 10 nm silica thickness (scale bar: 40 nm) (**a**) with absorbance spectra of silica coated gold nanoparticles in Milli-Q water without (**b**) and with fluorination (**c**) in perfluorohexane.

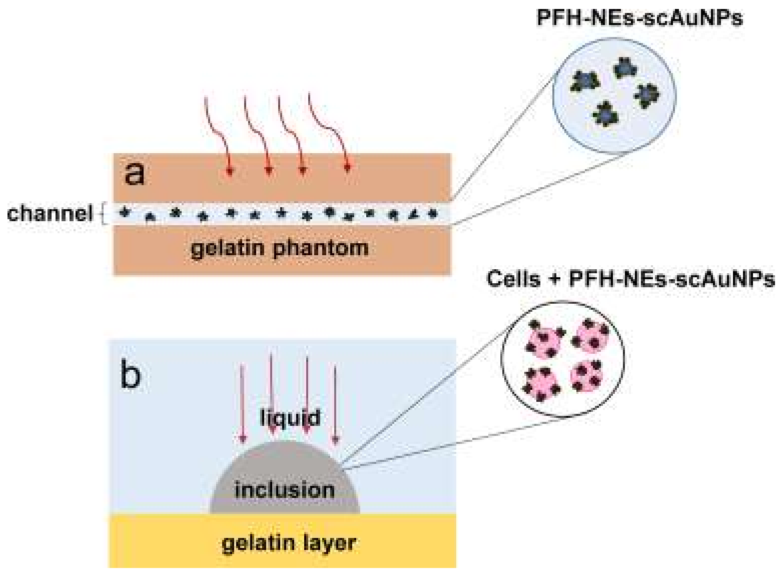

Figure S2. Schematic of experimental setup for imaging. Experimental setup for imaging NL US signals from PFH-NEs-scAuNPs in gelatin phantoms (**a**) and in inclusions (**b**) made up of cells and PFH-NEs-scAuNPs prior to vaporization of NEs into PFH bubbles.

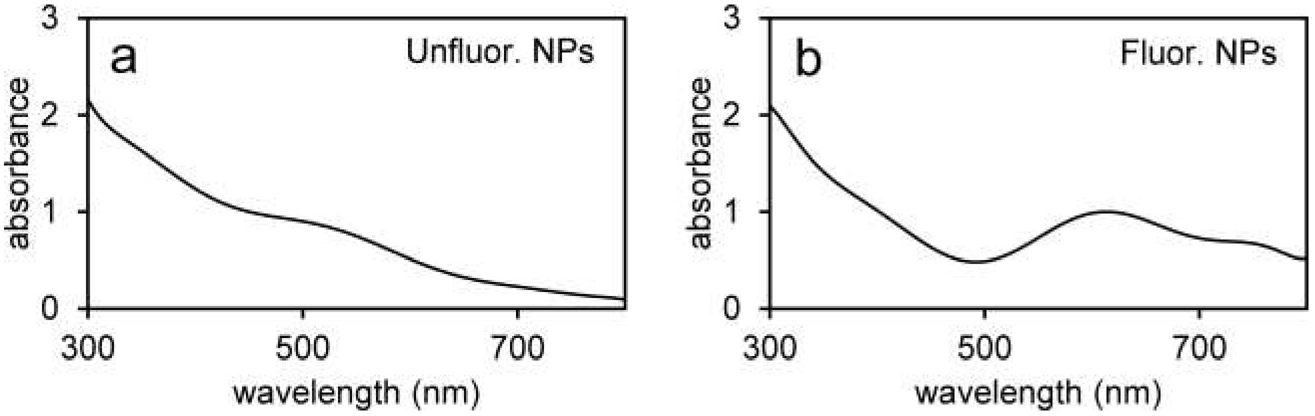

Figure S3. Absorption spectra of PFH-NEs-scAuNPs. Absorption spectra for unfluor. (**a**) and fluor. (**b**) NPs containing both PFH-NEs and scAuNPs (in Milli-Q water only).

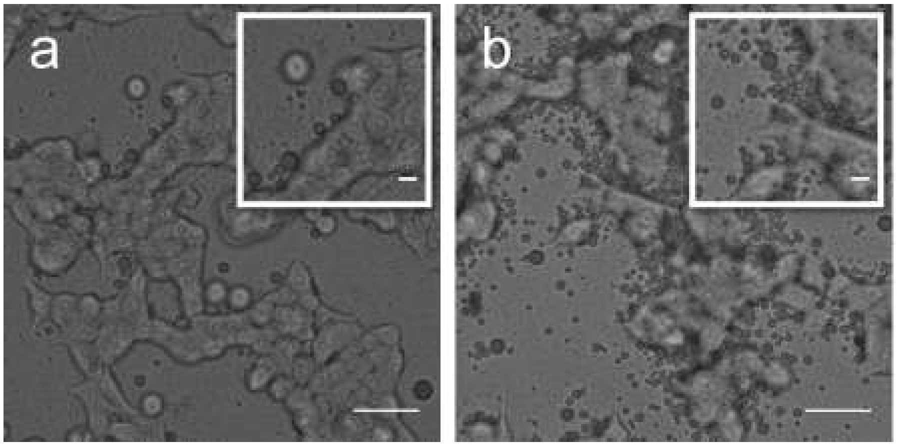

Figure S4. Brightfield images after vaporization from PFH-NEs-scAuNPs with MCF-7 cells. Brightfield images of PFH bubbles with cells after light illumination of unfluor. (**a**) and fluor. nanoparticles (**b**) (scale bar: 50 μm, inset scale bar: 10 μm).

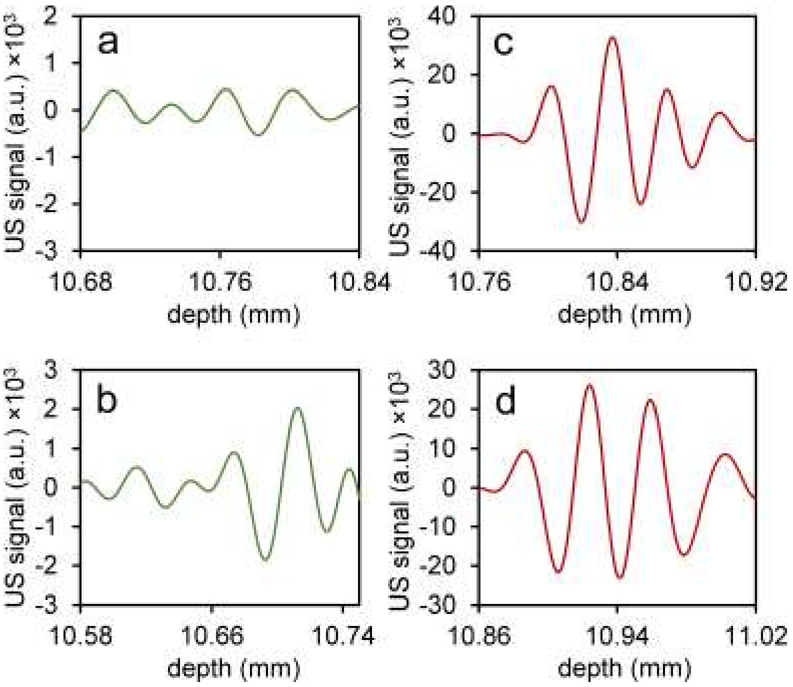

Figure S5. US signals from PFH-NEs and PFH bubbles. Representative US backscatter signals from NPs before (green) (**a,b**) and after vaporization of NEs from PFH bubbles (red) (**c,d**) from unfluor. (**a,c**) and fluor. (**b,d**) samples. Signals were collected from inclusions (channels) made using gelatin (see Supplementary Figure S2a). Note the differences in scale in the graphs before and after vaporization. Concentrations for unfluor. NPs was 2.5 mg/mL PFH-NEs with 0.37 µg/mL scAuNPs while for fluor. NPs it was 2.5 mg/mL PFH-NEs with 0.15 µg/mL scAuNPs.

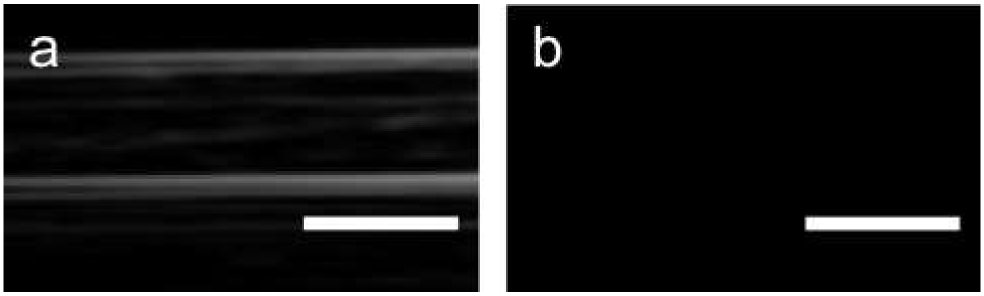

Figure S6. Ultrasound images from Milli-Q water. Representative B-mode (**a**) and nonlinear (**b**) ultrasound images for control inclusion with Milli-Q water only (at 37°C) after 10 s laser exposure at 680 nm (scale bar: 1 mm).

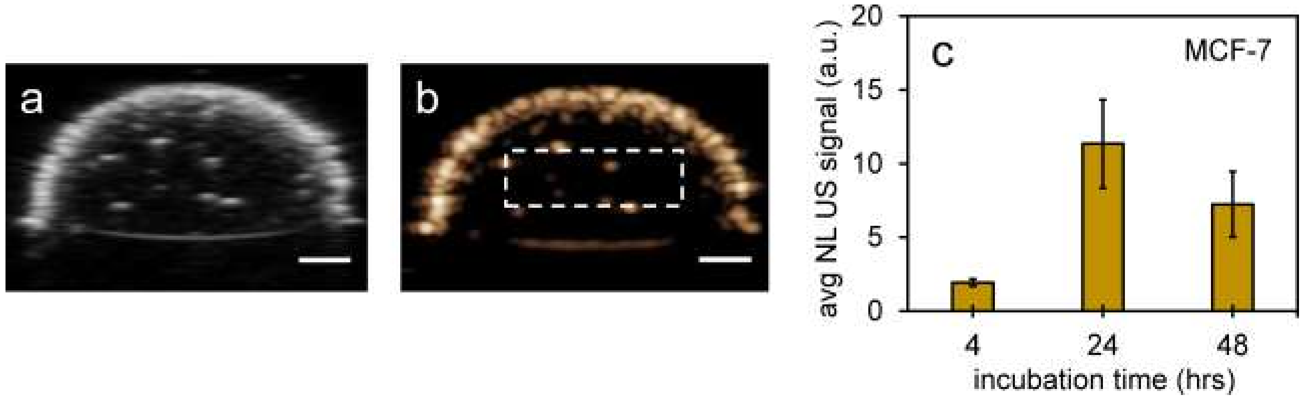

Figure S7. Nonlinear ultrasound imaging of MCF-7 cells only after laser exposure. Simultaneous images of MCF-7 cells only in inclusions from B-mode ultrasound (US) (**a**) and nonlinear ultrasound (NL US) (**b**) (scale bar: 1 mm) after 48 hrs incubation and 10 s laser exposure. NL US signals are shown for different incubation times (after laser exposure for 10 s at each time point) in (**c**) (all measurements at 37°C). All signals representing averaged gray scale values from three replicates measured from a rectangular region 3 mm × 1 mm at the center of the inclusion. Weak NL US signals were detected compared to NL US signals after vaporization of NEs from PFH-NEs-scAuNPs in MCF-7 cell inclusions which have signal values > 80 (see Figure 6). Each error bar represents standard deviation from three replicates.

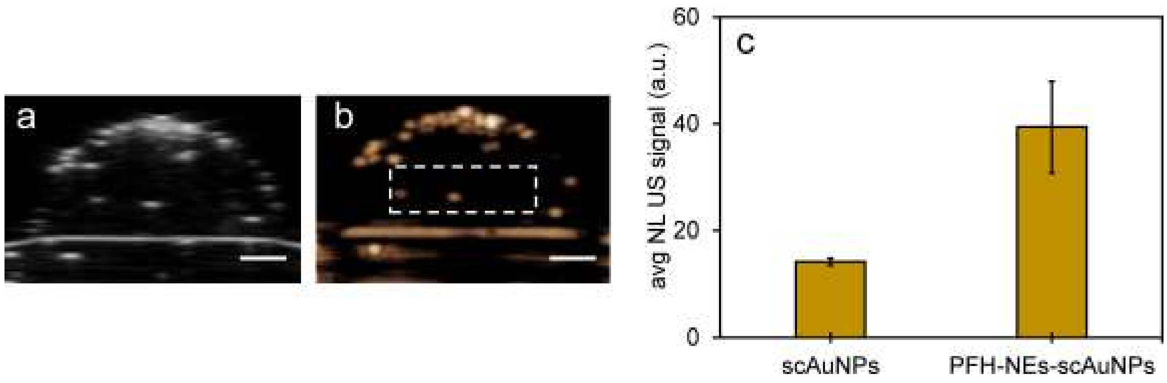

Figure S8. NL US signals from scAuNPs with MCF-7 cells after laser exposure. B-mode (**a**) and nonlinear US images (**b**) and the averaged intensity (**c**) after 24 hours incubation of cells with scAuNPs only and 10 s laser exposure (680 nm) compared with the averaged intensity from unfluor. sample from cells after vaporization (all measurements at 37°C) (scale bar: 1 mm) (same incubation time for particles). All signals represent averaged gray scale values from three replicates measured from a rectangular region 3 mm × 1 mm at the center of the inclusion. Weak NL US signals were detected compared to NL US signals after vaporization of NEs from PFH-NEs-scAuNPs in MCF-7 cell inclusions (Figure 6). Each error bar represents standard deviation from three replicates.

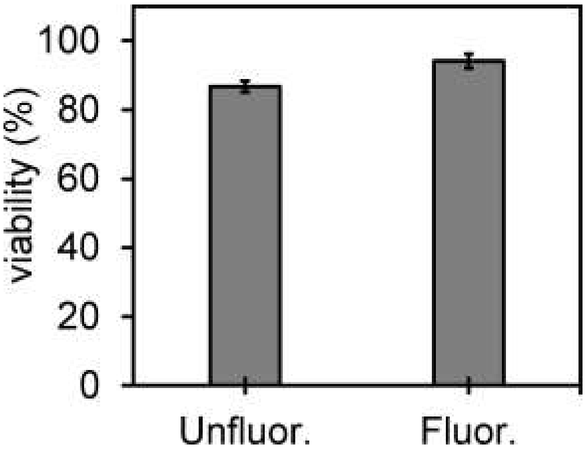

Figure S9. MCF-7 cells after treatment with blank PFH-NEs-scAuNPs. Cell viability of MCF-7 cells after 48 hours incubation at same concentrations used for NL US imaging of cancer cells.

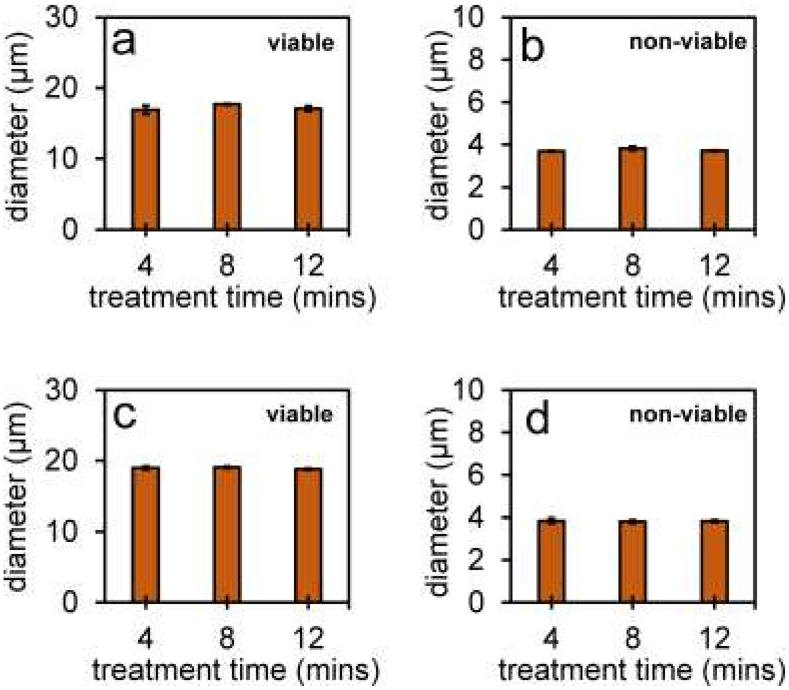

Figure S10. Cell size of PC-3 cells after treatment. Size of PC-3 prostate cancer cells after treatment with 680 nm laser excitation using NPs at the given time periods from unfluorinated (**a,b**) and fluorinated (**c,d**) samples. The averaged mean sizes (from three replicates) are shown from viable (**a,c**) and nonviable cells (**b,d**) determined using Vi-Cell XR Cell Viability Analyzer, which uses trypan blue to distinguish viable from nonviable cells. Each error bar represents standard deviation from three replicates.

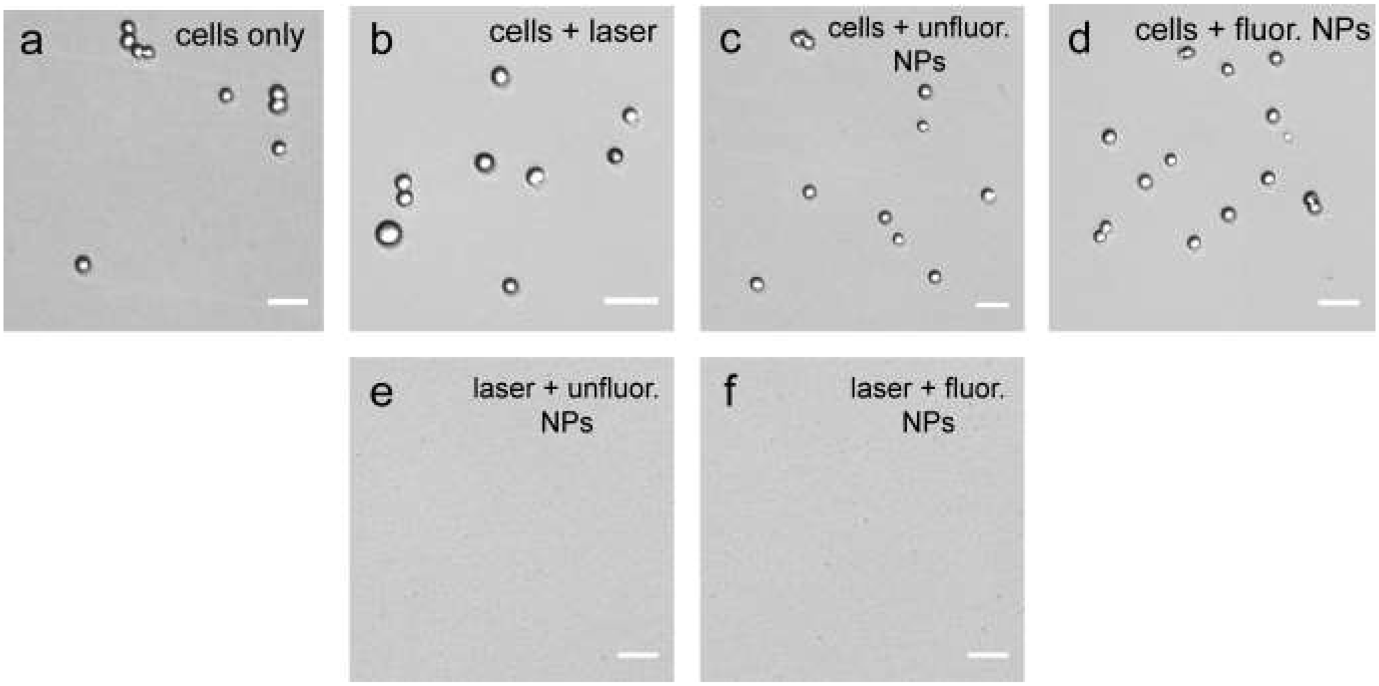

Figure S11. Images from control cells not treated with NPs and with combination with laser. Brightfield images of cells only (**a**), cells plus laser excitation (12 minutes, 680 nm) (**b**), cells plus unfluor. NPs (**c**) or fluor. NPs (**d**). As well as NPs were irradiated to determine the presence of any visible nanoparticles and bubbles (**e,f**). All controls were exposed to same laser irradiation conditions (where applicable) and washed using same procedure for determining viability in Figure 11 (scale bar: 50 μm). The controls for each treatment show that there are no non-viable cells, which are significantly detected only when cells are treated with both nanoparticles and laser excitation (seen in Figure 11).

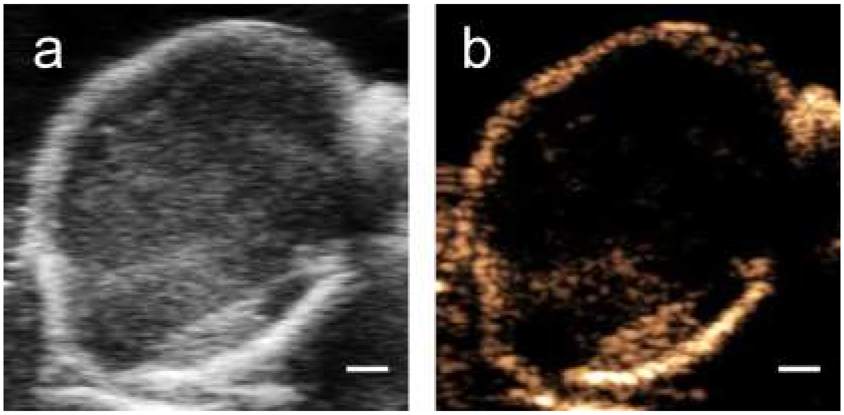

Figure S12. ***In vivo*** **NL US imaging with no contrast agents.** Linear ultrasound (**a**) and NL US (**b**) images and signals with no nanoparticles and bubbles within tumor. Images captured prior to injection of nanoparticles on day 0 (scale bar: 1 mm).

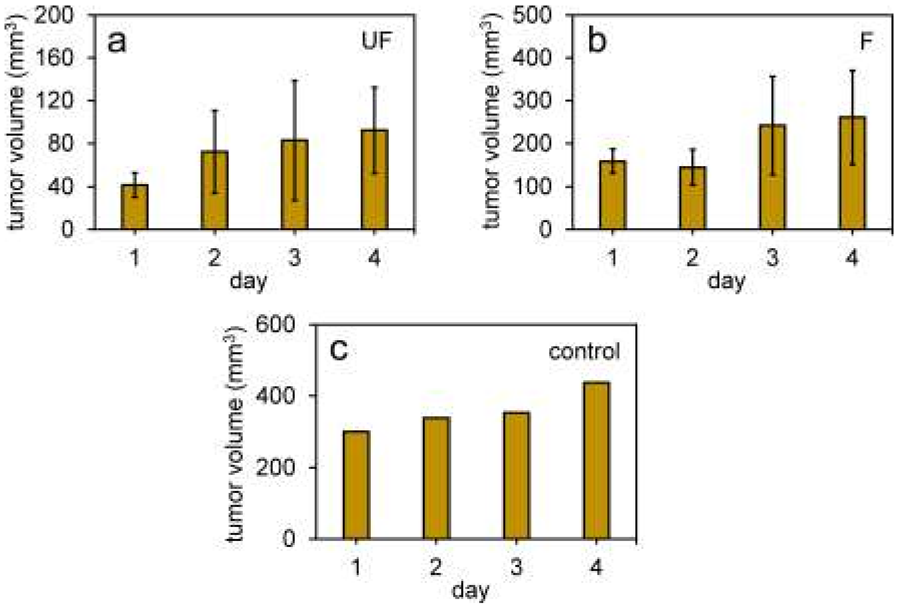

Figure S13. ***In vivo*** **therapeutic effect from PFH-NEs-scAuNPs.** Tumor volumes after laser treatment with PFH-NEs-scAuNPs from unfluorinated (UF) (**a**) and fluorinated (F) (**b**) nanoparticles with control (laser exposure only) shown (**c**). All tumors were treated with 5 minutes laser at 680 nm (using Vevo LAZR) on each day.
